# Inactivated SARS-CoV-2 Vaccine Booster Against Omicron Infection Among Quarantined Close Contacts

**DOI:** 10.1001/jamanetworkopen.2023.39507

**Published:** 2023-10-25

**Authors:** Di Liu, Siyang Feng, Feng Sha, Yuxue Liao, Xu Xie, Fang Huang, Dongfeng Kong, Zhen Zhang, Zhigao Chen, Nixuan Chen, Wei Gao, Tiejian Feng, Ziyi Zhao, Bingli Li, Ying Li, Fengcai Zhu, Zhirong Yang, Qiuying Lv, Zijian Feng, Jinling Tang

**Affiliations:** 1 Shenzhen Institute of Advanced Technology, Chinese Academy of Sciences, Shenzhen, China; 2 Shenzhen Center for Disease Control and Prevention, Shenzhen, China; 3 Research Department of Epidemiology and Public Health, University College London, London, United Kingdom; 4 Department of Vaccine Clinical Evaluation, Jiangsu Provincial Center for Disease Control and Prevention, Nanjing, China; 5 Primary Care Unit, Department of Public Health and Primary Care, School of Clinical Medicine, University of Cambridge, Cambridge, United Kingdom; 6 Division of Infectious Diseases, Chinese Center for Disease Control and Prevention, Beijing, China; 7 Division of Epidemiology, JC School of Public Health and Primary Care, Chinese University of Hong Kong, Hong Kong Special Administrative Region, China

## Abstract

**Importance:**

Assessment of additional protection of a booster dose with an inactivated SARS-CoV-2 vaccine is key to developing vaccination strategies for billions of people worldwide who have received the primary 2-dose regimen.

**Objective:**

To estimate the relative effectiveness of a booster dose of an inactivated SARS-CoV-2 vaccine against Omicron infection.

**Design, Setting, and Participants:**

This cohort study was conducted among primary close contacts without previous SARS-CoV-2 infection identified in Shenzhen, China, between February and October 2022. Multiple strict nucleic acid testing and symptom surveillance for SARS-CoV-2 infection were regularly conducted during the 7-day centralized plus 7-day home-based quarantine.

**Exposure:**

A booster with an inactivated SARS-CoV-2 vaccine vs no booster after receipt of the primary 2-dose inactivated SARS-CoV-2 vaccine regimen.

**Main Outcomes and Measures:**

The primary outcomes were overall, symptomatic, and asymptomatic infections. Secondary outcomes were length of incubation and level of cycle threshold values. All the outcomes were assessed during the quarantine period.

**Results:**

Among 119 438 eligible participants (mean [SD] age, 37.6 [12.0] years; 66 201 men [55.4%]), 86 251 (72.2%) received a booster dose of an inactivated SARS-CoV-2 vaccine and 33 187 (27.8%) did not. A total of 671 cases infected with Omicron BA.2 were confirmed (464 symptomatic and 207 asymptomatic), and no severe infection or death events were observed. At a median (IQR) duration of 111 (75 to 134) days after booster vaccination, the relative effectiveness of a booster was 32.2% (95% CI, 11.3% to 48.2%) for overall infection, 23.8% (95% CI, −8.2% to 46.4%) for symptomatic infection, and 43.3% (95% CI, 12.3% to 63.3%) for asymptomatic infection. The effectiveness against overall infection changed nonlinearly over time following booster vaccination: 44.9% (95% CI, 4.9% to 68.1%) within 60 days, 50.4% (95% CI, 23.7% to 67.7%) at 61 to 120 days, 29.1% (95% CI, −4.8% to 52.1%) at 121 to 180 days, and 19.4% (95% CI, −14.4% to 43.2%) after 180 days (nonlinear *P* = .03). The effectiveness did not vary significantly according to the interval between booster vaccination and completion of primary vaccination. There was no association of booster vaccination with incubation or cycle threshold values.

**Conclusions and Relevance:**

In this cohort study, a booster dose of an inactivated SARS-CoV-2 vaccine provided additional moderate protection against mild infection for 120 days after receipt, but more research is needed to determine the optimal timing of a booster and its effectiveness in preventing severe infection for a longer duration.

## Key Points

**Question:**

Does a booster dose with an inactivated SARS-CoV-2 vaccine provide additional protection against Omicron infection?

**Findings:**

In a cohort study involving regular nucleic acid testing of individuals in quarantine, 671 cases of Omicron BA.2 infection were confirmed among 119 438 close contacts without natural immunity. At a median of 111 days after booster vaccination, a booster dose provided effectiveness of 32.2% against overall infection on top of primary vaccination; the effectiveness was high (44.9%-50.4%) within 120 days of booster vaccination but waned afterward.

**Meaning:**

These findings suggest that a booster dose with an inactivated SARS-CoV-2 vaccine could provide additional moderate protection against Omicron infection within 120 days after receipt, but more research is needed to determine the optimal timing of a booster and its effectiveness in preventing severe infection for a longer duration.

## Teaser

This cohort study estimates the relative effectiveness of a booster dose of inactivated SARS-CoV-2 vaccine against Omicron infection.

## Introduction

Waning of the effectiveness of SARS-CoV-2 vaccines and the emergence of Omicron variants have stimulated efforts to scale up booster vaccination worldwide.^1,2^ Although the eligibility for booster vaccination has been expanded from high-risk people to the general population by World Health Organization and also in mainland China,^3,4^ few randomized clinical trials (RCTs) are available to confirm the effectiveness of booster vaccination against Omicron variants. Many general population observational studies were, thus, conducted. Both RCTs and observational studies, however, focused on the booster with mRNA and adenovirus vector vaccines,^2,5,6,7,8,9^ which may differ biologically from inactivated SARS-CoV-2 vaccines in terms of immunogenicity. Studies of inactivated SARS-CoV-2 vaccines are lacking for booster vaccination vs no booster vaccination, but the evidence is now still highly relevant for billions of people worldwide who have completed a 2-dose inactivated SARS-CoV-2 vaccine regimen.

Previous observational studies also had several important limitations. First, full coverage of nucleic acid testing for study participants was rare, and missing diagnoses may have caused severe biases to the effectiveness estimates.^10,11^ Second, the testing frequencies may have differed according to participants’ vaccination histories, which led to additional biases.^5,12^ Third, previous studies may have had selection bias by inevitably including some people who had no contact with patients with COVID-19 (ie, at no risk of infection at all) because of a lack of strict tracing of patients and their close contacts. Fourth, natural immunity may have contributed to vaccine effectiveness estimates, because many people in most Western countries would have been infected by other variants before Omicron-induced outbreaks. To overcome these limitations, complete detection of infections can be done only for individuals quarantined with strict nucleic acid testing. All close contacts of patients with COVID-19, who were representative of people at risk of the disease, can be identified only under regular mass nucleic acid testing and comprehensive epidemiological investigations. Data from China, where a large number of people had been quarantined under the zero-COVID policy, provided a rare valuable opportunity for addressing these issues.^13^ Such data are no longer available since the rigid COVID-19 restrictions in China were lifted in November 2022.

We, thus, conducted a large cohort study among quarantined close contacts to estimate the relative vaccine effectiveness (rVE) of a booster dose of an inactivated SARS-CoV-2 vaccine against Omicron-induced infection. The waning of the effectiveness of a booster over time was also assessed.

## Methods

Ethical approval for this cohort study was obtained from Shenzhen Center for Disease Control and Prevention, and no consent to participate was needed for this study because it retrospectively used anonymous data collected under the dynamic zero-COVID policy in China. We strictly followed the Strengthening the Reporting of Observational Studies in Epidemiology (STROBE) reporting guidelines for this cohort study.^14^

### Study Design and Population

This was a cohort study of close contacts of patients with confirmed SARS-CoV-2 infection identified in Shenzhen during an Omicron-induced outbreak from February to October 2022. Shenzhen is a metropolis with a population of 17 million local residents in Guangdong province. During the study period, Shenzhen residents were strongly recommended to attend the daily mass nucleic acid testing for SARS-CoV-2 infection so that they could obtain a negative result within 48 hours for entering public areas. In addition, Shenzhen has implemented comprehensive prevention and control measures to contain the outbreak under the zero-COVID policy, which helped identify all close contacts and infected people.^15,16^ When a new infection case was confirmed, all their close contacts were traced by comprehensive epidemiological investigations and the dates of last contact with the case were documented.^17^ We only included primary close contacts given that their exposure to SARS-CoV-2 was substantially higher than that of secondary close contacts (eTable 1 in Supplement 1).^17,18^ Under the 7 plus 7 quarantine policy (ie, 7-day centralized plus 7-day home-based quarantine), all close contacts were isolated and underwent strict nucleic acid testing on a regular basis (eTable 1 in Supplement 1). A close contact was eligible for our study if they had no previous SARS-CoV-2 infection and had been vaccinated with at least 2 doses of inactivated SARS-CoV-2 vaccines (ie, completing primary vaccination). Because booster vaccination was not recommended for children and adolescents, our study only included those aged 18 years or older. We excluded those with missing data for any covariate.

### Exposure Assessment

Vaccination status was obtained from the Shenzhen Government Immunization Program Information System, the repository of vaccination records on vaccine type, manufacturer, vaccination date, dose, and site for each individual. We verified the vaccination status by linking to the Guangdong Immunization Information System, which provided information on vaccination in other cities within Guangdong province outside Shenzhen. Vaccination status was divided into booster vaccination and no booster vaccination after the 2-dose primary regimen, with the third dose considered as a booster. The interval between the second and third doses was divided into 4 groups (ie, ≤180, 181-210, 211-240, and >240 days). The postbooster duration between the booster dose and the last contact with a SARS-CoV-2 infection case was also divided into 4 groups (ie, ≤60, 61-120, 121-180, and >180 days).

### Outcomes

The primary outcome was SARS-CoV-2 infection confirmed by nucleic acid testing conducted regularly during the 7 plus 7 quarantine (eTable 1 in Supplement 1). All confirmation assays were done by the Shenzhen Center for Disease Control and Prevention COVID-19 Emergency Laboratory. Once positive, final diagnosis was made by 2 infectious disease specialists (not coauthors of this article). Infected cases presenting any COVID-19 symptom during quarantine were classified as having symptomatic infection, and the rest were classified as having asymptomatic infection. Severe infection (eTable 1 in Supplement 1) and death were further recorded. Cycle threshold (Ct) was determined on the first day of a positive test (eTable 1 in Supplement 1). The incubation period was defined as the duration between the last contact date and date of diagnosis. Outcome information was collected during the quarantine and was linked to the vaccination records by using the unique identification number.

### Potential Confounder Covariates

On the basis of previous studies^5,7^ that reported risk factors for Omicron infection, potential baseline confounders adjusted in our analyses included age, sex, health care practitioner, diabetes, hypertension, chronic kidney disease, coronary heart disease, stroke, cancer, interval between the first and second dose, interval between the latest dose (third dose for the booster group and second dose for the no booster group) and last contact, and calendar weeks of last contact. Immunodeficiency and organ transplantation may also affect vaccine effectiveness, but neither was found in our study participants. Data on these variables were extracted from the China Information System for Disease Control and Prevention.^19^ All covariates were linked to the vaccination records by using the unique identification number.

### Statistical Analysis

Continuous variables were summarized as means and SDs, and categorical variables were summarized as counts and proportions. The differences in baseline characteristics by vaccination status were assessed using standardized mean difference, which is less sensitive to sample size than traditional tests in a large-scale study (a standardized mean difference >0.1 indicates between-group imbalance of baseline characteristics).^20^ Multivariable logistic regression was used to adjust for potential confounders and to estimate the odds ratio (OR) with 95% CI for overall, symptomatic, and asymptomatic infection, separately. Continuous variables (age, interval between the first and second doses, interval between the latest dose and last contact, and calendar weeks of last contact) were adjusted using restricted cubic splines function with 5 knots to capture possible nonlinear associations. Model 1 was adjusted for age, sex, health care practitioner, diabetes, hypertension, chronic kidney disease, stroke, cancer, interval between the first and second doses, and interval between the latest dose and last contact; model 2 was further adjusted for calendar weeks of last contact. Subgroup analyses was then performed by sex (male or female) and age (<60 or ≥60 years), with Wald test for the interaction. The rVE of booster vaccination over no booster vaccination was expressed as follows: (1 − adjusted OR) × 100% (eTable 1 in Supplement 1).

Furthermore, we used multivariable logistic regressions to explore the changes in the rVE of a booster over postbooster duration between the booster dose and the last contact or the interval between the second and third dose. Wald test and analysis of variances were performed to evaluate the linear and nonlinear trend, respectively, for these changes.^21^ We used multivariable linear regressions for assessing the association of the booster dose with Ct and incubation.

We performed 2 additional sensitivity analyses. To account for a potential time lag for developing immunological protection after vaccination, we conducted a sensitivity analysis restricted to those who had been vaccinated at least 14 days before the last contact date. Because the vaccination policy in China recommended that the interval between the third dose and second dose should be at least 180 days, we performed a sensitivity analysis by restricting the nonbooster group to those who received the second dose at least 180 days ago before the last contact date to make both groups more comparable in the interval.

All analyses were 2-sided, with *P *<* *.05 regarded as significant. Data cleaning and statistical analysis were performed using R statistical version 4.0.3 (R Project for Statistical Computing) and Stata statistical software version 15.0 (StataCorp).

## Results

**Figure 1.**
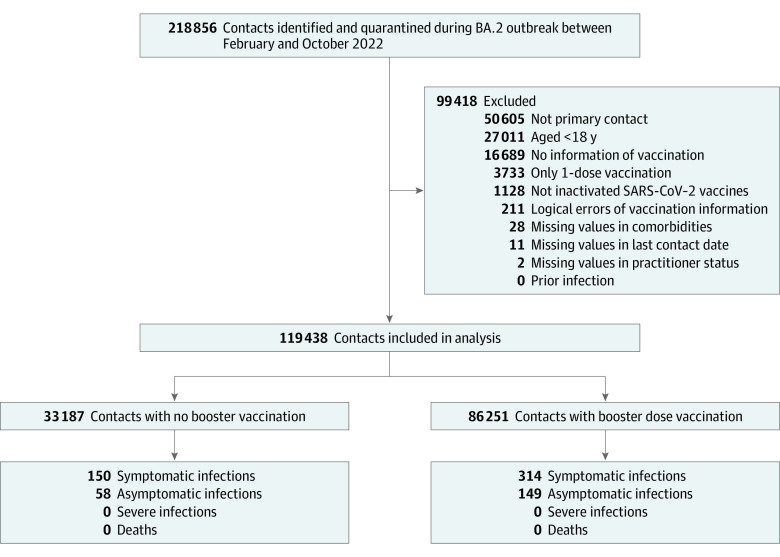
Flowchart of Participant Selection

Among a total of 119 438 eligible close contacts (66 201 men [55.4%]), 86 251 (72.2%) received a booster dose of an inactivated SARS-CoV-2 vaccine and 33 187 (27.8%) did not (Figure 1). Overall, the study population was young (mean [SD] age, 37.6 [12.0] years), although those with a booster dose were slightly older (Table 1). In total, 671 cases (0.56%) were detected, with 464 being symptomatic and 207 asymptomatic. All infections were caused by BA.2 and were confirmed by genome sequencing. No participants developed severe illness or died.

**Table 1.** Baseline Characteristics of Included Participants

Characteristics	Participants, No. (%)			SMD^a^
	Total (N = 119 438)	No booster group (n = 33 187)	Booster group (n = 86 251)	
Age, mean (SD), y	37.6 (12.0)	35.8 (12.8)	38.3 (11.7)	0.20
Sex				
Male	66 201 (55.4)	18 783 (56.6)	47 418 (55.0)	0.03
Female	53 237 (44.6)	14 404 (43.4)	38 833 (45.0)	
Underlying conditions				
Diabetes	2466 (2.1)	471 (1.4)	1995 (2.3)	0.07
Hypertension	2406 (2.0)	569 (1.7)	1837 (2.2)	0.03
Chronic kidney disease	151 (0.1)	32 (0.1)	119 (0.1)	0.01
Stroke	7 (<0.1)	1 (<0.1)	6 (<0.2)	0.006
Cancer	4983 (4.2)	849 (2.6)	4134 (4.9)	0.12
Interval between doses, mean (SD), d				
First and second dose	35.5 (25.2)	42.4 (42.0)	32.9 (13.2)	0.31
Second and third dose	218.9 (39.3)	NA	218.9 (39.3)	NA
Second dose and last contact	324.3 (96.2)	249.1 (88.5)	353.2 (82.5)	1.22
Third dose and last contact	134.3 (76.0)	NA	134.3 (76.0)	NA

Abbreviations: NA, not applicable; SMD, standardized mean difference.

^a^ SMD is shown as an absolute value, and a value of 0.1 or higher indicates imbalance in baseline characteristics.

Compared with no booster vaccination, the rVE of a booster was 31.8% (95% CI, 15.7% to 44.8%; model 1) and 32.2% (95% CI, 11.3% to 48.2%; model 2) for preventing overall infection, 43.5% (95% CI, 27.2% to 56.1%; model 1) and 23.8% (95% CI, −8.2% to 46.4%; model 2) for preventing symptomatic infection, and −3.7% (95% CI, −53.1% to 29.7%; model 1) and 43.3% (95% CI, 12.3% to 63.3%; model 2) for preventing asymptomatic infection at a median (IQR) duration of 111 (75-134) days after the booster vaccination. No statistically significant difference was observed for the rVE according to age or sex (Table 2).

**Table 2.** Vaccine Effectiveness of a Booster Dose vs No Booster Dose Against SARS-CoV-2 Infection

Outcome and subgroup	Cases, No./total No. (%)		Adjusted vaccine effectiveness, % (95% CI)	
	With no booster	With booster	Model 1^a^	Model 2^b^
Overall infection				
All participants	208/33 187 (0.63)	463/86 251 (0.54)	31.8 (15.7 to 44.8)	32.2 (11.3 to 48.2)
Age, y				
18-59	172/30 900 (0.56)	434/82 751 (0.52)	29.1 (9.3 to 42.7)	30.9 (7.2 to 48.6)
≥60	36/2287 (1.57)	29/3500 (0.83)	54.5 (16.9 to 75.1)	47.5 (−0.6 to 74.0)
*P* for interaction	NA	NA	.12	.25
Sex				
Male	115/18 783 (0.61)	223/47 418 (0.47)	41.6 (21.3 to 56.6)	31.2 (−2.5 to 53.8)
Female	93/14 404 (0.65)	240/38 833 (0.62)	20.9 (−8.2 to 40.8)	31.3 (0.9 to 52.3)
*P* for interaction	NA	NA	.13	.13
Symptomatic infection				
All participants	150/33 129 (0.45)	314/86 102 (0.36)	43.5 (27.2 to 56.1)	23.8 (−8.2 to 46.4)
Age, y				
18-59	126/30 854 (0.41)	295/82 612 (0.36)	41.0 (22.4 to 55.1)	28.0 (−5.0 to 50.6)
≥60	24/2275 (1.05)	19/3490 (0.54)	61.7 (19.5 to 81.7)	19.9 (−148 to 74.0)
*P* for interaction	NA	NA	.27	.50
Sex				
Male	82/18 750 (0.44)	151/47 346 (0.32)	57.4 (38.8 to 70.3)	32.5 (−12.4 to 59.5)
Female	68/14 379 (0.47)	163/38 756 (0.42)	25.5 (−6.8 to 48.0)	13.3 (−41.0 to 46.7)
*P* for interaction	NA	NA	.22	.26
Asymptomatic infection				
All participants	58/33 037 (0.18)	149/85 937 (0.17)	−3.7 (−53.1 to 29.7)	43.3 (12.3 to 63.3)
Age, y				
18-59	172/30 900 (0.56)	434/82 751 (0.52)	29.1 (9.3 to 42.7)	30.9 (7.2 to 48.6)
≥60	36/2287 (1.57)	29/3500 (0.83)	54.5 (16.9 to 75.1)	47.5 (−0.6 to 74.0)
*P* for interaction	NA	NA	.12	.25
Sex				
Male	33/18 701 (0.18)	72/47 267 (0.15)	−10.5 (−90.4 to 35.9)	32.1 (−30.9 to 64.7)
Female	25/14 336 (0.17)	77/38 670 (0.20)	5.6 (−64.5 to 45.8)	51.4 (13.5 to 72.7)
*P* for interaction	NA	NA	.35	.30

Abbreviation: NA, not applicable.

^a^ Model 1 was adjusted for age, sex, health care practitioner, diabetes, hypertension, chronic kidney disease, stroke, cancer, interval between the first and second doses, and interval between the latest dose and last contact.

^b^ Model 2 was also adjusted for calendar time of last contact.

**Figure 2.**
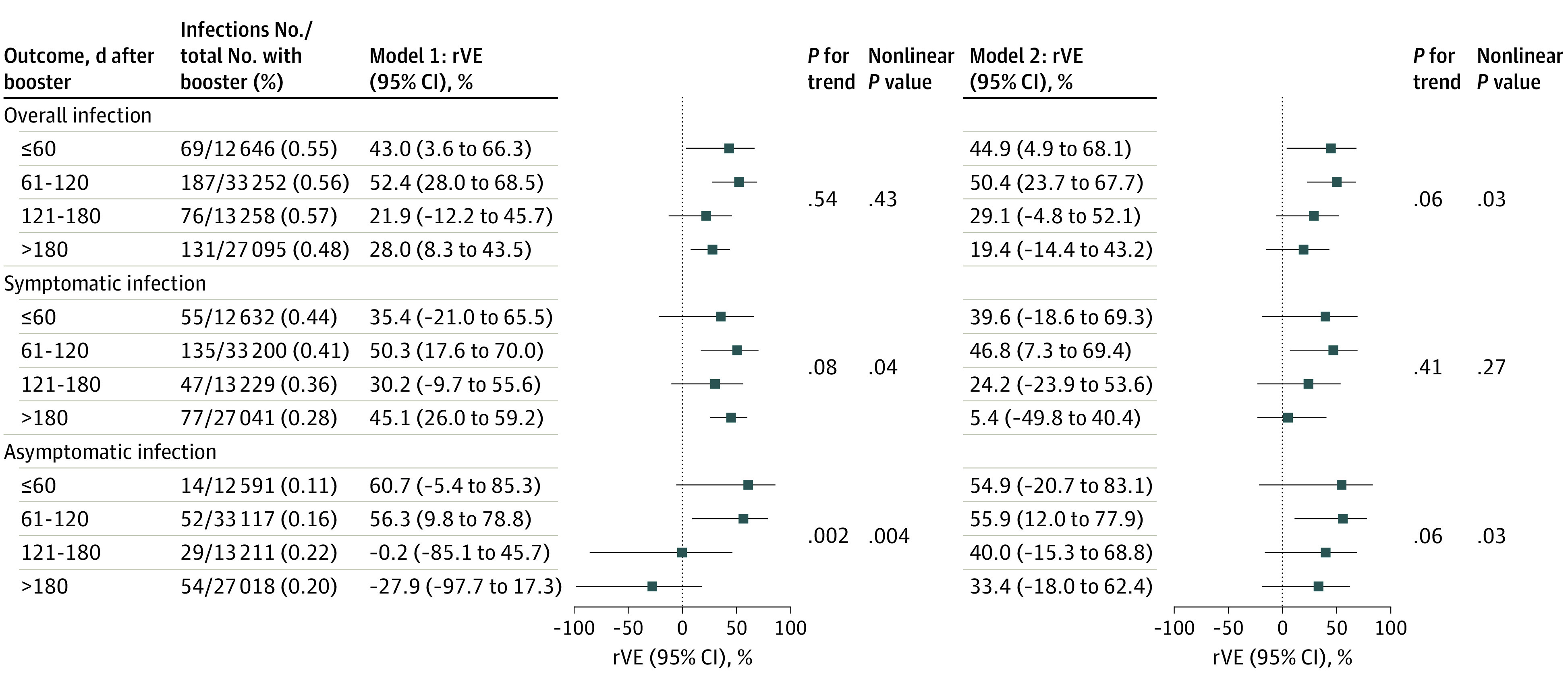
Changes in Relative Vaccine Effectiveness (rVE) of a Booster Dose Over Time Following Booster Vaccination Model 1 (left) was adjusted for age, sex, health care practitioner, diabetes, hypertension, chronic kidney disease, stroke, cancer, interval between the first and second doses, and interval between the latest dose and last contact. Model 2 (right) was further adjusted for calendar time of last contact. Test for linear trend was based on the median value of days assigned to the exposure groups.

The rVE of a booster dose against overall infection changed nonlinearly over time following booster vaccination, with 44.9% (95% CI, 4.9% to 68.1%) within 60 days after the booster dose, 50.4% (95% CI, 23.7% to 67.7%) at 61 to 120 days, 29.1% (95% CI, −4.8% to 52.1%) at 121 to 180 days, and 19.4% (95% CI, −14.4% to 43.2%) after 180 days in model 2 (*P* for trend = .06; nonlinear *P* = .03) (Figure 2). A similar nonlinear trend was also observed for rVE against asymptomatic infection but not for that against symptomatic infection.

**Figure 3.**
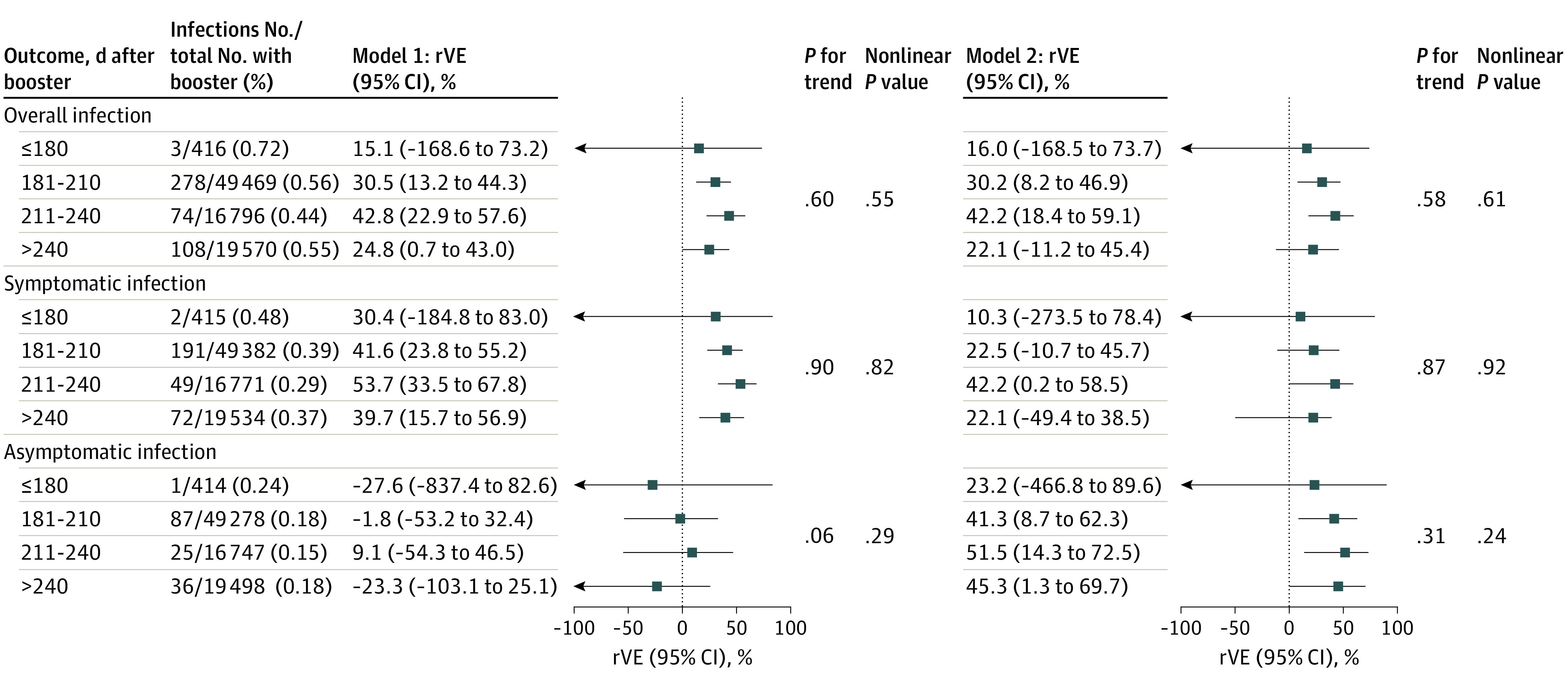
Relative Vaccine Effectiveness (rVE) of a Booster Dose According to Vaccination Interval Model 1 (left) was adjusted for age, sex, health care practitioner, diabetes, hypertension, chronic kidney disease, stroke, cancer, interval between the first and second doses, and interval between the latest dose and last contact. Model 2 (right) was further adjusted for calendar time of last contact. Test for linear trend was based on the median value of days assigned to the exposure groups.

The rVE of a booster dose in preventing overall infection, symptomatic infection, and asymptomatic infection did not vary significantly according to the interval between the booster vaccination and primary regimen (Figure 3). The booster dose was not associated with the length of incubation (mean difference, 0.25 days; 95% CI, −0.93 to 1.43 days; model 2) and the level of Ct values (mean difference, −0.12; 95% CI, −1.77 to 1.56; model 2) compared with no booster dose (eTable 2 in Supplement 1).

Sensitivity analysis accounting for potential time lag before development of immunological protection showed results similar to those of the main analyses (eTables 3, 4, 5, 6, and 7 in Supplement 1). In the sensitivity analysis, restricting the no booster group to those who were vaccinated with the second dose at least 180 days before last contact, the point estimates of rVE were largely unchanged, whereas the 95% CI became wider and statistical power became lower because of the smaller sample size included (eTables 8, 9, 10, 11, and 12 in Supplement 1).

## Discussion

In this cohort study with strict surveillance testing of SARS-CoV-2 infection, we found that a booster dose of an inactivated SARS-CoV-2 vaccine provided additional moderate protection against Omicron infection. The protection remained high within 120 days after booster vaccination but waned afterward. There was insufficient evidence to support that protection may vary according to the interval between the booster vaccination and primary regimen. We did not observe that booster vaccination was associated with the duration of incubation and level of Ct values.

### Comparisons With Previous Studies and Interpretations

Primary full vaccination is insufficiently effective in preventing infections, and its effectiveness also wanes rapidly over time,^22,23^ especially in preventing infection with Omicron variants.^24^ Thus, even a moderate 30% to 50% increase (depending on the time after booster vaccination) in rVE by a booster is useful in enhancing the effectiveness of primary vaccine regimen. For symptomatic infection or asymptomatic infection, however, our study suggested that such additional protection may be confounded by calendar time of the outbreak. This phenomenon was complicated because calendar time likely served as a proxy of a total effect of differences in the number of people at risk, the uptake of booster vaccination, and the intensity of nonpharmaceutical interventions during the outbreak.

In addition, there seems to be large heterogeneity in the effectiveness of booster vaccination vs no booster vaccination between studies.^25,26,27^ For example, our estimate was much lower than that of an mRNA vaccine found in an RCT conducted during the Delta-predominant period (95.3%; 95% CI, 89.5%-98.3%),^28^ but was closer to that of a recombinant protein vaccine (47.8%; 95% CI, 22.6%-64.7%) in another RCT^29^ conducted during the Omicron-predominant period. Furthermore, previous general population studies^25,27,30,31,32^ reported that the effectiveness of a booster dose of an inactivated SARS-CoV-2 vaccine against Omicron infection ranged between 10% and 60%, whereas that with mRNA vaccines ranged between 20% and 70%.^9,14,15^ These inconsistent findings suggest that the rVE of booster vaccination may be affected by type of vaccine, variant of the virus, population characteristics, and time after the booster vaccination.

Similar to primary vaccination, the effectiveness of a booster of an inactivated SARS-CoV-2 vaccine also waned. This is consistent with findings of other studies on mRNA vaccines,^2^ suggesting that waning is general feature of SARS-CoV-2 vaccines. For inactivated SARS-CoV-2 vaccines, a previous study^32^ suggested that there was no protection beyond 90 days after booster vaccination, but the protection remained significant within 120 days in our study. It is, however, unclear how long the protection will persist after booster vaccination. To maintain or enhance the effectiveness, a fourth dose or an Omicron-based vaccine may help, but further studies should confirm whether the fourth dose or the new formulation can really work for this purpose.^33,34^

Few studies have reported the association of interval between the booster vaccination and primary regimen with inactivated SARS-CoV-2 vaccines with the risk of SARS-CoV-2 infection. The effectiveness of a booster with mRNA vaccines against Omicron-induced infection was higher for the interval of more than 9 months than that of 5 to 9 months.^35,36^ However, there were insufficient data to show such a difference with inactivated SARS-CoV-2 vaccines in our study.

The Ct values and incubation period have been used as crude indicators of infectiousness.^37^ Previous studies^38,39,40^ showed that Ct values were significantly higher in cases vaccinated with a booster or primary 2-dose regimen compared with placebo. However, we did not observe that booster vaccination was associated with either the level of Ct values or length of incubation period, suggesting that a booster with an inactivated SARS-CoV-2 vaccine may not help reduce the infectiousness of Omicron variants.

### Strengths and Limitations

Our study minimized detection bias through strict, regular, nucleic acid testing for all participants. Because of regular mass nucleic acid testing across the city and comprehensive epidemiological investigations of patients with COVID-19 during the study period, all close contacts, who were representative of people at high risk of COVID-19, were identified and included in our study and their last contact dates and incubation period were ascertained. This kind of data is no longer available since the rigid COVID-19 restrictions in China were lifted in November 2022. In addition, the effectiveness estimates in our study population without previous infection would tease out the impact of natural immunity.

Our study also has several limitations. First, our findings are derived from relatively young close contacts without prior SARS-CoV-2 infection quarantined in Shenzhen under the zero-COVID policy and may not be generalized to different settings, such as older people, hybrid immunity, other types of SARS-CoV-2 vaccines, or other regions with a different level of natural immunity. Second, there was no progression to severe illness or death during the study period, and we were not able to investigate the effectiveness of booster vaccination against these outcomes. Third, the infection rate in this study was not as high as expected, mainly because loose criteria were used to define close contacts under the zero-COVID policy. Subgroup analysis could, thus, be insufficiently powered or even infeasible (eg, by comorbidities). Fourth, it is possible that some records of vaccine doses may be missing in the Shenzhen Immunization Program Information System for those vaccinated outside Shenzhen. Such missing data were likely for those with no booster but not those with a booster dose because, according to the booster vaccination policy in China, people could receive a maximum number of only 3 doses of inactivated SARS-CoV-2 vaccines during the study period. That is, those with data for only 2-dose vaccination may have received with a booster dose. Such misclassification may lead to underestimation of the rVE of a booster. For the same reason, people without any information on vaccination status were not necessarily those who were unvaccinated but could have been given with 1, 2, or 3 doses of vaccines outside Shenzhen, making it unsuitable to serve as a reference group in our study to estimate the absolute vaccine effectiveness of primary and booster immunization. Fifth, our observational study might have residual confounding for which we could not account, such as the relationship to the infected individuals, the time spent with them, contact environment, socioeconomic status, behaviors, and lifestyle factors. Sixth, because of the small number of SARS-CoV-2 infections and short postbooster duration, we were able to investigate only the early waning of rVE.

## Conclusions

In this cohort study, a booster with an inactivated SARS-CoV-2 vaccine provided additional protection against Omicron infection, highlighting the importance of achieving high coverage of booster doses. However, the protection waned after 120 days from booster vaccination. The booster dose may not affect the infectiousness of Omicron variants. More research is needed to determine the optimal time of a booster with an inactive SARS-CoV-2 vaccine and its effectiveness in preventing severe infection for a longer duration following booster vaccination. These findings are now particularly relevant for developing vaccination strategies for billions of people who have received a primary 2-dose regimen with inactivated SARS-CoV-2 vaccines.

## Notes

Supplement 1. **eTable 1.** Definitions of the Terms **eTable 2.** Length of Incubation and CT Value in Infected Patients by Booster Vaccination Status **eTable 3.** Sensitivity Analysis 1: Baseline Characteristics of Participants **eTable 4.** Sensitivity Analysis 1: Relative Effectiveness of a Booster Dose Against SARS-CoV-2 Infection **eTable 5.** Sensitivity Analysis 1: Changes in Relative Effectiveness of a Booster Dose Over Time Following Booster Vaccination **eTable 6.** Sensitivity Analysis 1: Relative Effectiveness of a Booster Dose According to Jab-Interval **eTable 7.** Sensitivity Analysis 1: Length of Incubation and CT Value in Infected Patients by Booster Vaccination Status **eTable 8.** Sensitivity Analysis 2: Baseline Characteristics of Participants **eTable 9.** Sensitivity Analysis 2: Relative Effectiveness of a Booster Dose Against SARS-CoV-2 Infection **eTable 10.** Sensitivity Analysis 2: Changes in Relative Vaccine Effectiveness of a Booster Dose Over Time Following Booster Vaccination **eTable 11.** Sensitivity Analysis 2: Relative Vaccine Effectiveness of a Booster Dose According to Jab-Interval **eTable 12.** Sensitivity Analysis 2: Length of Incubation and CT Value in Infected Patients by Booster Vaccination Status

Supplement 2.
Data Sharing Statement
