# Supplementary material for: Inactivated SARS-CoV-2 Vaccine Booster Against Omicron Infection Among Quarantined Close Contacts
Source: JAMA Netw Open. 2023 Oct 25;6(10):e2339507. doi: 10.1001/jamanetworkopen.2023.39507 (PMC10600580; doi:10.1001/jamanetworkopen.2023.39507)
Supplement: Supplement 1. — eTable 1. Definitions of the Terms eTable 2. Length of Incubation and CT Value in Infected Patients by Booster Vaccination Status eTable 3. Sensitivity Analysis 1: Baseline Characteristics of Participants eTable 4. Sensitivity Analysis 1: Relative Effectiveness of a Booster Dose Against SARS-CoV-2 Infection eTable 5. Sensitivity Analysis 1: Changes in Relative Effectiveness of a Booster Dose Over Time Following Booster Vaccination eTable 6. Sensitivity Analysis 1: Relative Effectiveness of a Booster Dose According to Jab-Interval eTable 7. Sensitivity Analysis 1: Length of Incubation and CT Value in Infected Patients by Booster Vaccination Status eTable 8. Sensitivity Analysis 2: Baseline Characteristics of Participants eTable 9. Sensitivity Analysis 2: Relative Effectiveness of a Booster Dose Against SARS-CoV-2 Infection eTable 10. Sensitivity Analysis 2: Changes in Relative Vaccine Effectiveness of a Booster Dose Over Time Following Booster Vaccination eTable 11. Sensitivity Analysis 2: Relative Vaccine Effectiveness of a Booster Dose According to Jab-Interval eTable 12. Sensitivity Analysis 2: Length of Incubation and CT Value in Infected Patients by Booster Vaccination Status [file jamanetwopen-e2339507-s001.pdf]

Supplemental Online Content

Liu D, Feng S, Sha F, et al. Inactivated SARS-CoV-2 vaccine booster against Omicron infection among quarantined close contacts. *JAMA Netw Open*. 2023;6(10):e2339507. doi:10.1001/jamanetworkopen.2023.39507

eTable 1. Definitions of the Terms
eTable 2. Length of Incubation and CT Value in Infected Patients by Booster Vaccination Status
eTable 3. Sensitivity Analysis 1: Baseline Characteristics of Participants
eTable 4. Sensitivity Analysis 1: Relative Effectiveness of a Booster Dose Against SARS-CoV-2 Infection
eTable 5. Sensitivity Analysis 1: Changes in Relative Effectiveness of a Booster Dose Over Time Following Booster Vaccination
eTable 6. Sensitivity Analysis 1: Relative Effectiveness of a Booster Dose According to Jab-Interval
eTable 7. Sensitivity Analysis 1: Length of Incubation and CT Value in Infected Patients by Booster Vaccination Status
eTable 8. Sensitivity Analysis 2: Baseline Characteristics of Participants
eTable 9. Sensitivity Analysis 2: Relative Effectiveness of a Booster Dose Against SARS-CoV-2 Infection
eTable 10. Sensitivity Analysis 2: Changes in Relative Vaccine Effectiveness of a Booster Dose Over Time Following Booster Vaccination
eTable 11. Sensitivity Analysis 2: Relative Vaccine Effectiveness of a Booster Dose According to Jab-Interval
eTable 12. Sensitivity Analysis 2: Length of Incubation and CT Value in Infected Patients by Booster Vaccination Status

This supplemental material has been provided by the authors to give readers additional information about their work.

© 2023 Liu D et al. *JAMA Network Open.*

eTable 1. Definitions of the Terms

Term	Definition/Explanation
Primary close contact	That is "a person who has unprotected close contact with an individual with confirmed or suspected COVID-19 within two days before illness onset or with an asymptomatic carrier within two days before sampling".
Secondary close contact	That is "a person who has unprotected close contact (being in the same enclosed environment for living, work, a gathering, or entertainment) with a primary close contact".
"7+7" quarantine policy	Under the "7+7" quarantine policy (7-day centralized plus 7-day home-based quarantine), all close contacts were isolated for 7-day medical monitoring in quarantine centers that were remodeled from small and medium-sized hotels or hostels, during which nucleic acid testing was conducted on all at day 1, 2, 3, 5 and 7 of the quarantine. When all tests in the centers were negative, they were sent back for further 7-day home-based quarantine, during which nucleic acid testing was conducted on day 1, 4 and 7 of the quarantine.
SARS-CoV-2 infection confirmed by nucleic acid testing	Real-time fluorescent quantitative reverse transcription polymerase chain reaction (RFQ-RT-PCR) was performed to test dual-target (ORF1ab, N gene) using SARS-CoV-2 Nucleic acid detection kit according to the manufacturer's instructions for nucleic acids extracted from oropharyngeal swabs. Cycle threshold (CT) was determined on the first day of a positive test.
Severe infection	All infected cases found during quarantine, regardless of severity, should be sent immediately to designated hospitals for medical care and monitoring, allowing to observe and record the whole clinical course of infection including severe infection and death. Severe COVID-19 cases encompass instances where patients require oxygen supplementation, admission to intensive care units, or succumb to the disease. The criteria for classifying an illness as severe are as follows: (a) Respiratory distress, defined as a respiration rate (RR) equal to or exceeding 30 breaths per minute (BPM), (b) Resting oxygen saturation at or below 93%, (c) Arterial partial pressure of oxygen to fraction of inspired oxygen ratio (PaO2/FiO2) at or below 300 mmHg. Furthermore, cases exhibiting chest imaging revealing significant lesion progression within 24–48 hours—greater than 50% change—should also be categorized as severe.
Relative vaccine effectiveness	Effectiveness of a booster dose (3rd dose) compared to no booster dose (2nd dose).
Absolute vaccine effectiveness	Effectiveness of a booster dose (3rd dose) compared to no vaccination (0 dose).

© 2023 Liu D et al. *JAMA Network Open.*

eTable 2. Length of Incubation and CT Value in Infected Patients by Booster Vaccination Status

**Outcomes**	**Booster group**	**No booster group**	**Adjusted mean difference (95%CI)**	
			**Model 1**	**Model 2**
Both infection (n)	463	208		
Length of incubation, mean (SD)	2.46 (5.18)	2.29 (2.14)	0.51 (-0.45, 1.47)	0.25 (-0.93, 1.43)
CT value, mean (SD)	26.49 (6.32)	26.01 (6.28)	0.98 (-0.38, 2.34)	-0.12 (-1.77, 1.56)
Symptomatic infection (n)	314	150		
Length of incubation, mean (SD)	2.61 (5.70)	2.38 (2.28)	1.13 (-0.19, 2.45)	0.69 (-1.05, 2.43)
CT value, mean (SD)	25.74 (6.19)	25.76 (6.28)	0.004 (-1.69, 1.70)	-0.20 (-2.45, 2.05)
Asymptomatic infection (n)	149	58		
Length of incubation, mean (SD)	2.13 (3.86)	2.05 (1.71)	-0.14 (-1.54, 1.26)	-0.54 (-2.10, 1.03)
CT value, mean (SD)	28.07 (6.33)	26.81 (6.28)	1.65 (-0.92, 4.23)	-0.47 (-3.29, 2.34)

Model 1: adjusted for age, gender, care provider, diabetes, hypertension, chronic kidney disease, stroke, cancer, time interval between the first and second doses, and time interval between the latest dose and last contact.

Model 2: additionally adjusted for calendar time of last contact.

CI: confidence interval; CT: cycle threshold; MD: mean difference; SD: standard deviation.

© 2023 Liu D et al. *JAMA Network Open.*

eTable 3. Sensitivity Analysis 1: Baseline Characteristics of Participants

**Characteristics**	**Total (N=117,419), No. (%)**	**No booster group (n= 33,043), No. (%)**	**Booster group (n= 84,376), No. (%)**	**SMD**
Age (years), mean (SD)	37.6 (12.0)	35.8 (12.8)	38.3 (11.7)	0.19
Sex				0.03
Male	65,208 (55.4)	18,693 (56.6)	46,335 (55.0)	
Female	52,211 (44.6)	14,350 (43.4)	38,041 (45.0)	
Diabetes	2,451 (2.1)	469 (1.4)	1,982 (2.3)	0.07
Hypertension	2,386 (2.0)	569 (1.7)	1,817 (2.2)	0.03
Chronic kidney disease	151 (0.1)	32 (0.1)	119 (0.1)	0.01
Stroke	7 (0.006)	1 (0.003)	6 (0.007)	0.006
Cancer	4,964 (4.2)	847 (2.6)	4,117 (4.9)	0.12
Days between 1st and 2nd dose, mean (SD)	35.5 (25.2)	42.1 (41.2)	32.9 (13.1)	0.30
Days between 2nd and 3rd dose, mean (SD)	228.2 (46.8)	NA	228.2 (46.8)	NA
Days between 2nd dose and last contact, mean (SD)	326.0 (95.7)	250.2 (87.3)	355.6 (81.5)	1.25
Days between 3rd dose and last contact, mean (SD)	107.2 (95.4)	NA	107.2 (95.4)	NA

This sensitivity analysis was conducted restricting to individuals who had been vaccinated at least 14 days before the last contact date.

NA: not applicable; No.: number of participants; SD: standard deviation; SMD: standardized mean difference (shown as an absolute value, >=0.1 indicates imbalance in baseline characteristics).

© 2023 Liu D et al. *JAMA Network Open.*

eTable 4. Sensitivity Analysis 1: Relative Effectiveness of a Booster Dose Against SARS-CoV-2 Infection

**Outcome and subgroup**	**Case/Total (%) with no booster**	**Case/Total (%) with booster**	**Adjusted rVE (95% CI), %**	
			**Model 1**	**Model 2**
Overall infection				
All participants	207/33,043 (0.63)	456/84,376 (0.54)	31.5 (15.4, 44.6)	31.0 (9.5, 47.4)
Age				
18-59 years	171/30,770 (0.56)	430/81,051 (0.53)	27.5 (8.8, 42.4)	30.0 (5.9, 48.0)
60+ years	36/2,273 (1.58)	26/3,325 (0.78)	54.7 (16.4, 75.5)	44.7 (-14.8, 73.3)
P-interaction			0.07	0.17
Sex				
Male	114/18,693 (0.61)	218/46,335 (0.47)	41.5 (20.9, 56.6)	29.2 (-6.0, 52.7)
Female	93/14,350 (0.65)	238/38,041 (0.63)	19.9 (-8.3, 40.7)	30.8 (0.2, 52.0)
P-interaction			0.13	0.12
Symptomatic infection				
All participants	149/32,985 (0.45)	310/84,230 (0.37)	43.1 (26.6, 55.9)	22.2 (-10.9, 45.3)
Age				
18-59 years	125/30,724 (0.41)	293/80,914 (0.36)	40.4 (21.5, 54.7)	26.0 (-8.1, 49.4)
60+ years	24/2,261 (1.06)	17/3,316 (0.51)	61.1 (17.5, 81.6)	17.5 (-157.7, 73.6)
P-interaction			0.19	0.38
Sex				
Male	81/18,660 (0.43)	149/46,266 (0.32)	56.9 (38.1, 70.0)	30.8 (-15.8, 58.7)
Female	68/14,325 (0.47)	161/37,964 (0.42)	25.5 (-6.8, 48.0)	12.0 (-43.6, 46.0)
P-interaction			0.25	0.29
Asymptomatic infection				
All participants	58/32,894 (0.18)	146/84,066 (0.17)	-3.2 (-52.3, 30.1)	43.1 (11.6, 63.3)
Age				
18-59 years	47/30,764 (0.15)	137/81,152 (0.17)	-12.7 (-73.5, 26.7)	37.7 (-2.3, 62.1)
60+ years	11/2,130 (0.52)	9/2,914 (0.31)	43.6 (-61.3, 80.3)	58.1 (-20.0, 85.4)
P-interaction			0.19	0.23
Sex				
Male	33/18,612 (0.18)	69/46,186 (0.15)	-8.8 (-88.2, 37.1)	31.0 (-34.7, 64.7)
Female	25/14,282 (0.18)	77/37,880 (0.20)	5.2 (-65.0 45.5)	51.0 (12.9, 72.5)
P-interaction			0.29	0.24

This sensitivity analysis was conducted restricting to those who had been vaccinated at least 14 days before the last contact date.

Model 1: adjusted for age, gender, care provider, diabetes, hypertension, chronic kidney disease, stroke, cancer, time interval between the first and second doses, and time interval between the latest dose and last contact.

Model 2: additionally adjusted for calendar time of last contact.

CI: confidence interval; rVE: relative vaccine effectiveness.

© 2023 Liu D et al. *JAMA Network Open.*

eTable 5. Sensitivity Analysis 1: Changes in Relative Effectiveness of a Booster Dose Over Time Following Booster Vaccination

Outcome and duration (days)	Case/Total (%) with booster	Adjusted rVE (95% CI), %	
		**Model 1**	**Model 2**
Overall infection			
<=60	62/10,771 (0.58)	43.8 (3.5, 67.3)	45.4 (4.6, 68.8)
61-120	187/33,252 (0.56)	50.9 (25.2, 67.8)	48.1 (19.4, 66.5)
121-180	76/13,258 (0.57)	22.5 (-11.3, 46.1)	29.1 (-6.6, 51.3)
>180	131/27,095 (0.48)	28.0 (8.3, 43.5)	19.4 (-15.3, 43.1)
P-trend		0.57	0.08
P-nonlinear		0.46	0.04
Symptomatic infection			
<=60	51/10,760 (0.47)	35.3 (-23.2, 66.0)	37.9 (-24.1, 68.9)
61-120	135/33,200 (0.41)	47.2 (11.2, 68.6)	42.8 (-1.2, 67.7)
121-180	47/13,229 (0.36)	30.1 (-9.8, 55.5)	22.5 (-26.9, 52.7)
>180	77/27,041 (0.28)	45.2 (26.1, 59.3)	6.4 (-48.9, 41.2)
P-trend		0.11	0.41
P-nonlinear		0.06	0.27
Asymptomatic infection			
<=60	11/10,720 (0.10)	64.0 (-2.1, 87.3)	60.4 (-11.3, 85.9)
61-120	52/33,117 (0.16)	57.8 (12.8, 79.5)	56.0 (11.4, 78.0)
121-180	29/13,211 (0.22)	3.0 (-79.0, 47.4)	39.8 (-15.4, 68.6)
>180	54/27,018 (0.20)	-28.4 (-98.6, 17.1)	31.7 (-21.4, 61.6)
P-trend		0.002	0.04
P-nonlinear		0.006	0.02

This sensitivity analysis was conducted restricting to those who had been vaccinated at least 14 days before the last contact date.

Model 1: adjusted for age, gender, care provider, diabetes, hypertension, chronic kidney disease, stroke, cancer, time interval between the first and second doses, and time interval between the latest dose and last contact.

Model 2: additionally adjusted for calendar time of last contact.

Test for linear trend was based on the median value of days assigned to the exposure groups.

CI: confidence interval; rVE: relative vaccine effectiveness.

© 2023 Liu D et al. *JAMA Network Open.*

eTable 6. Sensitivity Analysis 1: Relative Effectiveness of a Booster Dose According to Jab-Interval

Outcome and jab-interval (days)	Case/Total (%) with booster	Adjusted rVE (95% CI), %	
		Model 1	Model 2
Overall infection			
<=180	3/407 (0.74)	14.0 (-172.4, 72.8)	13.9 (-175.5, 73.1)
181-210	276/48,894 (0.56)	30.2 (12.8, 44.1)	28.8 (6.2, 46.0)
211-240	72/16,271 (0.44)	43.0 (23.0, 57.8)	41.7 (17.4, 58.9)
>240	105/18,804 (0.56)	24.2 (-0.3, 42.8)	20.1 (-14.5, 44.2)
P-trend		0.71	0.70
P-nonlinear		0.64	0.71
Symptomatic infection			
<=180	2/406 (0.49)	29.6 (-188.1, 82.8)	7.8 (-284.3, 77.9)
181-210	189/48,807 (0.39)	41.4 (23.5, 55.1)	20.8 (-13.4, 44.7)
211-240	48/16,247 (0.30)	53.6 (33.1, 67.8)	34.4 (-2.0, 57.8)
>240	71/18,770 (0.38)	38.0 (13.1, 55.8)	0.8 (-57.6, 35.6)
P-trend		0.88	0.84
P-nonlinear		0.78	0.88
Asymptomatic infection			
<=180	1/405 (0.25)	-30.3 (-855.5, 82.2)	22.0 (-475.9, 89.4)
181-210	87/49,278 (0.18)	-2.6 (-54.6, 31.8)	40.7 (7.6, 62.0)
211-240	25/16,747 (0.15)	10.7 (-52.5, 47.7)	52.6 (15.5, 73.4)
>240	36/19,498 (0.18)	-18.5 (-96.7, 28.6)	47.1 (3.3, 71.0)
P-trend		0.09	0.33
P-nonlinear		0.28	0.24

This sensitivity analysis was conducted restricting to those who had been vaccinated at least 14 days before the last contact date.

Model 1: adjusted for age, gender, care provider, diabetes, hypertension, chronic kidney disease, stroke, cancer, tine interval between the first and second doses, and time interval between the latest dose and last contact.

Model 2: additionally adjusted for calendar time of last contact.

Test for linear trend was based on the median value of days assigned to the exposure groups.

CI: confidence interval; rVE: relative vaccine effectiveness.

© 2023 Liu D et al. *JAMA Network Open.*

eTable 7. Sensitivity Analysis 1: Length of Incubation and CT Value in Infected Patients by Booster Vaccination Status

Outcomes	Booster group	No booster group	Adjusted mean difference (95%CI)	
			**Model 1**	**Model 2**
Both infection (n)	456	207		
Length of incubation, mean (SD)	2.45 (5.21)	2.29 (2.14)	0.49 (-0.48, 1.46)	0.20 (-0.99, 1.40)
CT value, mean (SD)	26.53 (6.31)	26.06 (6.29)	0.96 (-0.41, 2.32)	-0.08 (-1.76, 1.59)
Symptomatic infection (n)	310	149		
Length of incubation, mean (SD)	2.61 (5.73)	2.39 (2.29)	1.12 (-0.21, 2.45)	0.66 (-1.09, 2.42)
CT value, mean (SD)	25.81 (6.16)	25.77 (6.30)	0.02 (-1.68, 1.71)	-0.08 (-1.76, 1.59)
Asymptomatic infection (n)	146	58		
Length of incubation, mean (SD)	2.12 (3.88)	2.05 (1.71)	-0.15 (-1.56, 1.26)	-0.59 (-2.17, 0.97)
CT value, mean (SD)	28.05 (6.37)	26.81 (6.28)	1.51 (-1.07, 4.11)	-0.44 (-3.28, 2.40)

This sensitivity analysis was conducted restricting to those who had been vaccinated at least 14 days before the last contact date.

Model 1: adjusted for age, gender, care provider, diabetes, hypertension, chronic kidney disease, stroke, cancer, time interval between the first and second doses, and time interval between the latest dose and last contact.

Model 2: additionally adjusted for calendar time of last contact.

CI: confidence interval; CT: cycle threshold; MD: mean difference; SD: standard deviation.

© 2023 Liu D et al. *JAMA Network Open.*

eTable 8. Sensitivity Analysis 2: Baseline Characteristics of Participants

**Characteristics**	**Total (N=113,876), No. (%)**	**No booster group (n= 27,625), No. (%)**	**Booster group (n= 86,251), No. (%)**	**SMD**
Age (years), mean (SD)	37.5 (11.8)	35.1 (11.7)	38.3 (11.7)	0.27
Gender				0.04
Male	63,119 (55.4)	15,701(56.8)	47,418 (55.0)	
Female	50,757 (44.6)	11,924 (43.2)	38,833 (45.0)	
Diabetes	2,345 (2.1)	350 (1.3)	1,995(2.3)	0.08
Hypertension	2,284 (2.0)	447 (1.6)	1,837 (2.2)	0.04
Chronic kidney disease	140 (0.1)	21 (0.1)	119 (0.1)	0.02
Stroke	7 (0.006)	1 (0.003)	6 (0.007)	0.005
Cancer	4,816 (4.2)	682 (2.5)	4,134 (4.9)	0.12
Days between 1st and 2nd dose, mean (SD)	33.4 (15.3)	34.9 (20.5)	32.9 (13.2)	0.12
Days between 2nd and 3rd dose, mean (SD)	218.9 (39.3)	NA	218.9 (39.3)	NA
Days between 2nd dose and last contact, mean (SD)	334.4 (85.9)	275.9 (68.1)	353.2 (82.5)	1.02
Days between 3rd dose and last contact, mean (SD)	134.3 (76.0)	NA	134.3 (76.0)	NA

This sensitivity analysis was conducted restricting no booster group to individuals who had been vaccinated at least 180 days before the last contact date.

NA: not applicable; No.: number of participants; SD: standard deviation; SMD: standardized mean difference (shown as an absolute value, >=0.1 indicates imbalance in baseline characteristics).

© 2023 Liu D et al. *JAMA Network Open.*

eTable 9. Sensitivity Analysis 2: Relative Effectiveness of a Booster Dose Against SARS-CoV-2 Infection

Outcome and subgroup	Case/Total (%) with no booster	Case/Total (%) with booster	Adjusted rVE (95% CI), %	
			**Model 1**	**Model 2**
Overall infection				
All participants	169/27,625 (0.61)	463/86,251 (0.54)	32.7 (13.6, 47.6)	25.3 (-8.2, 48.4)
Age				
18-59 years	149/26,418 (0.56)	434/82,751 (0.52)	28.8 (7.3, 45.3)	23.6 (-12.3, 48.0)
60+ years	20/1,207 (1.66)	29/3,500 (0.83)	56.5 (-12.2, 83.2)	52.0 (-119.2, 89.5)
P-interaction			0.09	0.22
Sex				
Male	97/15,701 (0.62)	223/47,418 (0.47)	47.7 (25.6, 63.3)	31.0 (-15.3, 58.8)
Female	72/11,924 (0.60)	240/38,833 (0.62)	12.2 (-25.6, 38.6)	18.2 (-40.2. 52.2)
P-interaction			0.09	0.09
Symptomatic infection				
All participants	126/27,582 (0.46)	314/86,102 (0.36)	49.9 (31.9, 63.1)	22.0 (-24.9, 51.3)
Age				
18-59 years	111/26,380 (0.42)	295/82,612 (0.36)	45.6 (24.9, 60.5)	21.8 (-27.3, 51.9)
60+ years	15/1,202 (1.25)	19/3,490 (0.54)	79.7 (24.4, 94.6)	36.2 (-448.9, 92.6)
P-interaction			0.12	0.34
Sex				
Male	70/15,674(0.45)	151/47,346 (0.32)	64.6 (44.6, 77.3)	35.4 (-25.2, 66.7)
Female	56/11,908 (0.47)	163/38,756 (0.42)	29.7 (-7.7, 54.2)	5.0 (-87.7, 51.9)
P-interaction			0.26	0.27
Asymptomatic infection				
All participants	43/27,499 (0.16)	149/85,937 (0.17)	-27.6 (-101.4, 19.2)	30.9 (-29.5, 63.1)
Age				
18-59 years	38/26,307 (0.14)	434/82,751 (0.52)	-29.9 (-112.0, 20.6)	27.2 (-40.9, 62.4)
60+ years	5/1192 (0.42)	29/3,500 (0.83)	-50.1 (-549.5, 65.3)	58.6 (-283.8, 95.6)
P-interaction			0.37	0.37
Sex				
Male	27/15,631 (0.17)	72/47,267 (0.15)	-9.7 (-102.4, 40.6)	29.6 (-66.5, 69.4)
Female	16/11,868 (0.13)	77/38,670 (0.20)	-50.9 (-201.2, 24.4)	34.4 (-67.1, 74.2)
P-interaction			0.15	0.14

This sensitivity analysis was conducted restricting no booster group to individuals who had been vaccinated at least 180 days before the last contact date.

Model 1: adjusted for age, gender, care provider, diabetes, hypertension, chronic kidney disease, stroke, cancer, time interval between the first and second doses, and time interval between the latest dose and last contact.

Model 2: additionally adjusted for calendar time of last contact.

CI: confidence interval; rVE: relative vaccine effectiveness.

© 2023 Liu D et al. *JAMA Network Open.*

eTable 10. Sensitivity Analysis 2: Changes in Relative Vaccine Effectiveness of a Booster Dose Over Time Following Booster Vaccination

Outcome and duration (days)	Case/Total (%) with booster	Adjusted rVE (95% CI), %	
		Model 1	Model 2
Overall infection			
<=60	69/12,646 (0.55)	73.1 (18.1, 91.1)	62.8 (-14.8, 88.0)
61-120	187/33,252 (0.56)	73.6 (28.3, 90.3)	63.3 (-2.1, 86.8)
121-180	76/13,258 (0.57)	46.2 (2.2, 70.4)	45.8 (-3.6, 71.7)
>180	131/27,095 (0.48)	32.7 (13.4, 47.6)	21.2 (-15.1, 46.0)
P-trend		0.56	0.25
P-nonlinear		0.47	0.26
Symptomatic infection			
<=60	55/12,632 (0.44)	87.3 (49.0, 96.8)	75.6 (0.1, 94.1)
61-120	135/33,200 (0.41)	88.0 (56.8, 96.6)	76.2 (11.7, 93.6)
121-180	47/13,229 (0.36)	66.9 (28.3, 84.7)	54.8 (-3.3, 80.2)
>180	77/27,041 (0.28)	49.6 (31.4, 63.0)	12.2 (-43.4, 46.2)
P-trend		0.01	0.38
P-nonlinear		0.12	0.25
Asymptomatic infection			
<=60	14/12,591(0.11)	-2.9 (-614.9, 85.2)	32.7 (-389.5, 85.9)
61-120	52/33,117 (0.16)	-15.5 (-494.7, 77.6)	30.0 (-264.9, 86.6)
121-180	29/13,211 (0.22)	-37.5 (-258.0, 47.2)	34.4 (-89.4, 77.3)
>180	54/27,018 (0.20)	-26.5 (-100.3, 20.1)	31.0 (-29.9, 63.4)
P-trend		0.17	0.03
P-nonlinear		0.51	0.14

This sensitivity analysis was conducted restricting no booster group to individuals who had been vaccinated at least 180 days before the last contact date.

Model 1: adjusted for age, gender, care provider, diabetes, hypertension, chronic kidney disease, stroke, cancer, time interval between the first and second doses, and time interval between the latest dose and last contact.

Model 2: additionally adjusted for calendar time of last contact.

Test for linear trend was based on the median value of days assigned to the exposure groups.

CI: confidence interval; rVE: relative vaccine effectiveness.

© 2023 Liu D et al. *JAMA Network Open.*

eTable 11. Sensitivity Analysis 2: Relative Vaccine Effectiveness of a Booster Dose According to Jab-Interval

**Outcome and jab-interval (days)**	**Case/Total (%) with booster**	**Adjusted rVE (95% CI), %**	
		**Model 1**	**Model 2**
Overall infection			
<=180	3/416 (0.72)	16.0 (-168.0, 73.7)	6.1 (-207.8, 71.4)
181-210	278/49,469 (0.56)	31.3 (11.1, 46.8)	21.2 (-14.1, 45.6)
211-240	74/16,796 (0.44)	43.9 (22.1, 59.6)	35.2 (0.1, 58.0)
>240	108/19,570 (0.55)	25.8 (-0.1, 45.7)	10.7 (-41.7, 43.7)
P-trend		0.60	0.44
P-nonlinear		0.55	0.57
Symptomatic infection			
<=180	2/415 (0.48)	38.8 (-153.2, 85.2)	6.3 (-302.2, 78.2)
181-210	191/49,382 (0.39)	48.0 (28.7, 62.0)	18.0 (-30.8, 48.6)
211-240	49/16,771 (0.30)	59.2 (39.0, 72.7)	32.7 (-15.2, 60.7)
>240	72/19,534 (0.37)	47.0 (22.3, 63.8)	0.2 (-75.3, 43.1)
P-trend		0.90	0.90
P-nonlinear		0.83	0.95
Asymptomatic infection			
<=180	1/414 (0.24)	-59.9 (-855.5, 82.2)	6.4 (-625.1, 87.9)
181-210	87/49,278 (0.18)	-25.1 (-54.6, 31.8)	28.6 (-35.2, 62.3)
211-240	25/16,747 (0.15)	-11.3 (-52.5, 47.7)	40.5 (-26.6, 72.1)
>240	36/19,498 (0.18)	-55.6 (-96.7, 28.6)	27.5 (-66.0, 68.3)
P-trend		0.04	0.21
P-nonlinear		0.28	0.26

This sensitivity analysis was conducted restricting no booster group to individuals who had been vaccinated at least 180 days before the last contact date.

Model 1: adjusted for age, gender, care provider, diabetes, hypertension, chronic kidney disease, stroke, cancer, time interval between the first and second doses, and time interval between the latest dose and last contact.

Model 2: additionally adjusted for calendar time of last contact.

Test for linear trend was based on the median value of days assigned to the exposure groups.

CI: confidence interval; rVE: relative vaccine effectiveness.

© 2023 Liu D et al. *JAMA Network Open.*

eTable 12. Sensitivity Analysis 2: Length of Incubation and CT Value in Infected Patients by Booster Vaccination Status

**Outcomes**	**Booster group**	**No booster group**	**Adjusted mean difference (95%CI)**	
			**Model 1**	**Model 2**
Both infection (n)	463	169		
Length of incubation, mean (SD)	2.46 (5.18)	2.24 (2.18)	1.06 (-0.09, 2.22)	1.34 (-0.43, 3.12)
CT value, mean (SD)	26.49 (6.32)	26.34 (6.38)	0.71 (-0.90, 2.31)	-1.47 (-3.92, 0.97)
Symptomatic infection (n)	314	126		
Length of incubation, mean (SD)	2.61 (5.70)	2.32 (2.32)	1.65 (0.16, 3.15)	2.45 (-0.03, 4.93)
CT value, mean (SD)	25.74 (6.19)	26.11 (6.37)	-0.28 (-2.18, 1.62)	-1.23 (-4.39, 1.94)
Asymptomatic infection (n)	149	43		
Length of incubation, mean (SD)	2.13 (3.86)	2.02 (1.71)	0.37 (-1.49, 2.24)	-0.21 (-2.69, 2.28)
CT value, mean (SD)	28.07 (6.33)	27.03 (6.46)	2.00 (-1.34, 5.34)	-2.68 (-7.04, 1.68)

This sensitivity analysis was conducted restricting no booster group to individuals who had been vaccinated at least 180 days before the last contact date.

Model 1: adjusted for age, gender, care provider, diabetes, hypertension, chronic kidney disease, stroke, cancer, time interval between the first and second doses, and time interval between the latest dose and last contact.

Model 2: additionally adjusted for calendar time of last contact.

CI: confidence interval; CT: cycle threshold; MD: mean difference; SD: standard deviation.

© 2023 Liu D et al. *JAMA Network Open.*
