# Supplementary material for: Inactivated SARS-CoV-2 Vaccine Booster Against Omicron Infection Among Quarantined Close Contacts
Source: JAMA Netw Open. 2023 Oct 25;6(10):e2339507. doi: 10.1001/jamanetworkopen.2023.39507 (PMC10600580; doi:10.1001/jamanetworkopen.2023.39507)
Supplement: Supplement 2. — Data Sharing Statement [file jamanetwopen-e2339507-s002.pdf]

Data Sharing Statement

Liu. Inactivated SARS-CoV-2 Vaccine Booster Against Omicron Infection Among Quarantined Close Contacts. *JAMA Netw Open*. Published October 25, 2023.
doi:10.1001/jamanetworkopen.2023.39507

Data
Data available: No

Additional Information
Explanation for why data not available: The analyses for this study were based on anonymous data collected under the dynamic Zero-COVID policy in China. Due to data privacy regulations, no individual-level data used in our study are publicly available.
